# Correlation of five secretory proteins with the nasopharyngeal carcinoma metastasis and the clinical applications

**DOI:** 10.18632/oncotarget.14725

**Published:** 2017-01-18

**Authors:** Ya Tao, Kun Wang, Zhen Chen, Lu Long, Qiong Wu, Facai Cui, Lepan Zhu, Manlin Xiang, Yuan Jiang, Yunlai Liang, Shiyang Qiu, Zhiqiang Xiao, Bin Yi

**Affiliations:** ^1^ Department of Clinical Laboratory, Xiangya Hospital, Central South University, Changsha, Hunan, China; ^2^ Department of Clinical Laboratory, Hunan Cancer Hospital, Changsha, Hunan, China; ^3^ The Higher Educational Key Laboratory for Cancer Proteomics and Translational Medicine of Hunan, Xiangya Hospital, Central South University, Changsha, Hunan, China

**Keywords:** nasopharyngeal carcinoma, metastasis, secretory proteins, serum tumor biomarker, ADAMTSL4

## Abstract

In our previous study, five different secretory proteins, including GSN, ADAMTSL4, CALR, PPIA and TXN, have been identified to be associated with the nasopharyngeal carcinoma (NPC) metastasis. In this work, the 5 proteins were further investigated. Bioinformatics analysis suggested that they might play an important role in the process of NPC development. Western blotting analysis showed that all of these 5 targets could be secreted into extracellular by both high metastatic NPC 5-8F cells and non-metastatic NPC 6-10B cells. Except for GSN, the expressions of ADAMTSL4, CALR, PPIA and TXN proteins in extracts of the 5-8F and 6-10B cells were significantly different (*P* < 0.05). Thus, the expressions of these 4 differentially expressed proteins were further tested in a cohort of NPC tissue specimens. The results indicated that the expression levels of ADAMTSL4 and TXN were highly correlated with the lymph node and distant metastasis (*P*<0.05) in NPC patients. Moreover, Enzyme-linked immunosorbent assay (ELISA) was used to investigate the concentrations of the ADAMTSL4 and TXN in serum specimens of NPC patients. The results revealed that serum ADAMTSL4 expression level was closely correlated with lymph node metastasis and clinical stage (*P*<0.05) in NPC patients, and it was able to discriminate metastasis NPC from non-metastasis NPC with a sensitivity of 75.6% and a specificity of 64.7%. The present data show for the first time that the ADAMTSL4 and TXN may be novel and potential biomarkers for predicting the NPC metastasis.Furthermore, the serum ADAMTSL4 could be a potential serum tumor biomarker for prognosis of NPC.

## INTRODUCTION

Nasopharyngeal carcinoma (NPC) is a common head and neck cancer in southern China and Southeast Asia [1–3]. Due to the indistinct symptoms of the disease and relatively deep location of the nasopharynx [4], most NPC patients are diagnosed at late stages with the risk of developing cervical nodal and/or distant metastasis detrimental for patient outcome. Meanwhile, the molecular mechanism of NPC metastasis is not yet well-defined. To develop better diagnosis and treatment approaches, it is important to identify the proteins related to the NPC metastasis, especially the proteins from human serum or other body fluids.

Recently, many studies have shown that secretory proteins (secretome) can be a fundamental source for biomarkers discovery [5–7]. The secretome, defined by Tjalsma and his colleagues [8] in 2000, is used to describe proteins secreted/shed from cells, tissues or organs. It plays an important role in growth, angiogenesis, invasion and metastasis of cancer-cells. Through screening and identifying the secretome in tumor cells, many researchers are trying to discover tumor biomarkers and therapeutic targets from human serum or other body fluids using proteomics technologies, and have received great achievements [9–11].

Isobaric tags for relative and absolute quantitation (iTRAQ) is a multifunctional proteomics technology developed by American ABI company in 2004 [12]. In our previous study [13], to identify metastasis-related proteins in NPC, iTRAQ-tagging combined with 2D LC-MS/MS analysis was performed to identify the differentially expressed proteins (DEPs) in high metastatic NPC 5-8F cells and non-metastatic NPC 6-10B cells, and a total of 101 DEPs were found. Through Uniprot and Pubmed search, 12 targets (GSN, PPIA, RAN, SQSTM1, TRIM29, TXN, CALR, DIABLO, PDIA4, SPTBN1, ADAMTSL4 and PTRF) were chosen for further verification. Quantitative Reverse transcriptase Polymerase chain reaction (qRT-PCR) was performed to test their expression in 5-8F and 6-10B cells. As a result, the expression changes of mRNAs for five targets were consistent with the findings in MS analysis, including A disintegrin and metalloproteinase with thrombospondin motifs-like protein 4 (ADAMTSL4), Calreticulin (CALR), Gelsolin (GSN), Peptidyl-prolyl cis-trans isomerase A (PPIA) and Thioredoxin (TXN). Thus, these five secretory proteins could be further investigated as the candidates. In addition, although being studied in several cancers [14–18], these five proteins have not been researched as secretory proteins in NPC.

Therefore, in this study, ADAMTSL4, CALR, GSN, PPIA and TXN were investigated as the candidate secretory proteins to verify the specific secreted proteins correlated to the metastasis of NPC and identify novel serum biomarkers for the early detection of NPC.

## RESULTS

### GO analysis, miRNAs analysis, pathway and Protein-protein interaction (PPI) analysis of the 5 DEPs(GSN, ADAMTSL4, CALR, PPIA, TXN) in NPC development process

According to GO analysis, these 5 DEPs were dividing into three groups(Supplementary Figure S1A). In the biological process, some of these were associated with apoptosis, cell redox homeostasis. In the cellular component, the proteins mainly participated in cytoplasmic and extracellular region.Moreover, some of these proteins were binding proteins with the molecular functions analysis, like the protein binding, unfolded protein binding and calcium ion binding. Supplementary Figrue S1B showed the miRNAs of genes corresponding to the 5 DEPs.Furthermore, the GenMAPP pathway analysis showed that the 5 DEPs significantly enriched in unfolded protein binding signaling pathway, cell proliferation pathway, cell motility pathway, etc. They may play an important role in the process of nasopharyngeal carcinoma development as Supplementary Figrue S1C described. PPI networks of differentially-expressed genes were shown in Supplementary Figrue S1D.

### Identification of whether GSN, ADAMTSL4, CALR, PPIA and TXN can be secreted by 6-10B and 5-8F cells by using Western blotting analysis

**Figure 1:**
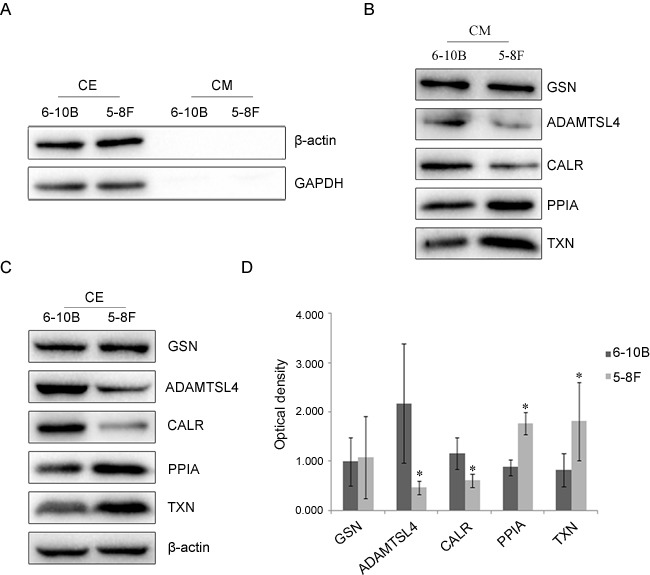
Expressional changes of GSN, ADAMTSL4, CALR, PPIA and TXN in NPC cells **A**. Western blotting analysis of antibodies against β-actin and GAPDH proteins in cell extracts (CE) and conditioned media (CM) from NPC-6-10B and NPC-5-8F cell lines.**B**. Western blotting analysis of GSN, ADAMTSL4, CALR, PPIA and TXN in CM from 6-10B and 5-8F cell lines.**C**. Expression capabilities of the five candidate proteins in CE from 5-8F and 6-10B cells. **D.** Expression levels of the candidate five proteins in 5-8F and 6-10B cells determined by densitometric analysis. β-actin is used as the internal loading control. Bars = means ± SD; *P < 0.05.

To check the qualities of the cell extracts (CE) and conditioned media (CM), Western blotting analysis was conducted to examine the presence of two abundant cytoplasmic proteins: β-actin and glyceraldehyde-3-phosphate dehydrogenase (GAPDH). As shown in Figure 1A, both proteins were clearly detected in cell extracts, but barely observed in conditioned media, which indicated that proteins recovered in conditioned media were not the result of cell death. Then, it was identified that the proteins of GSN, ADAMTSL4, CALR, PPIA and TXN were expressed by NPC-6-10B and NPC-5-8F cells in conditioned media (Figure 1B).

### Western blotting and immunohistochemitry analyses of the five candidate proteins expression in NPC cells and tissue specimens

To identify the result of our previous study, Western blotting and immunohistochemitry analyses were used to check the expression of the five candidate proteins in NPC cells and tissue specimens. The result of Western blotting analysis (Figure 1C and 1D) showed that the expression of ADAMTSL4 and CALR was much lower in 5-8F cells than that in 6-10B cells (*P* < 0.05), while the expression of PPIA and TXN was significantly higher in 5-8F cells than that in 6-10B cells (*P* < 0.05), which are well consistent with the results of MS and qRT-PCR analysis. The expression of GSN showed no significant difference(*P* > 0.05)in these two NPC cells.

**Figure 2:**
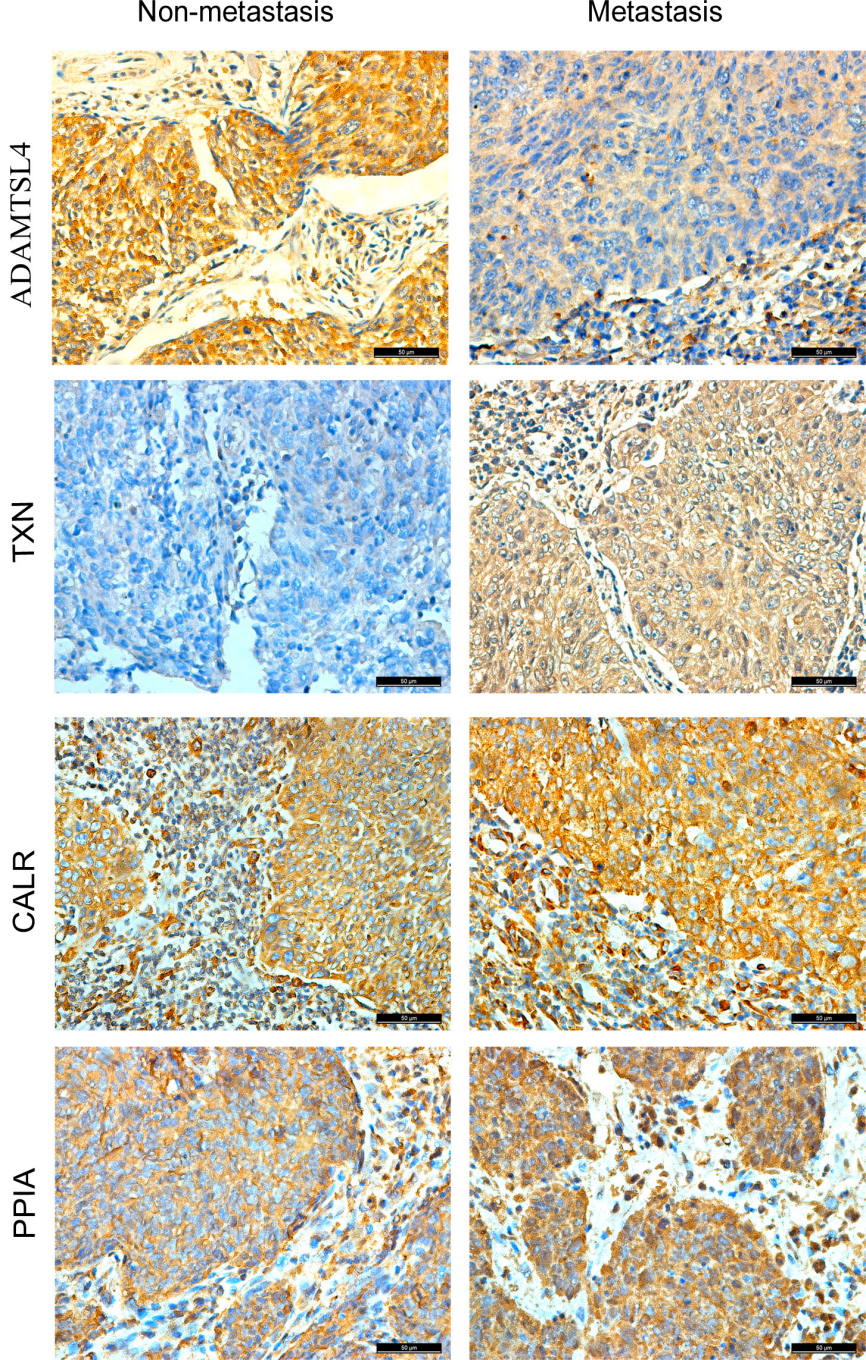
Expressional changes of ADAMTSL4, CALR, PPIA and TXN in NPC tissues Immunohistochemitry was further performed to validate the expression of ADAMTSL4, TXN, CALR and PPIA in non-metastasis and metastasis NPC tissue specimens. IHC staining patterns of each target protein from two Representative cases. Scale bar, 50 μm.

**Figure 3:**
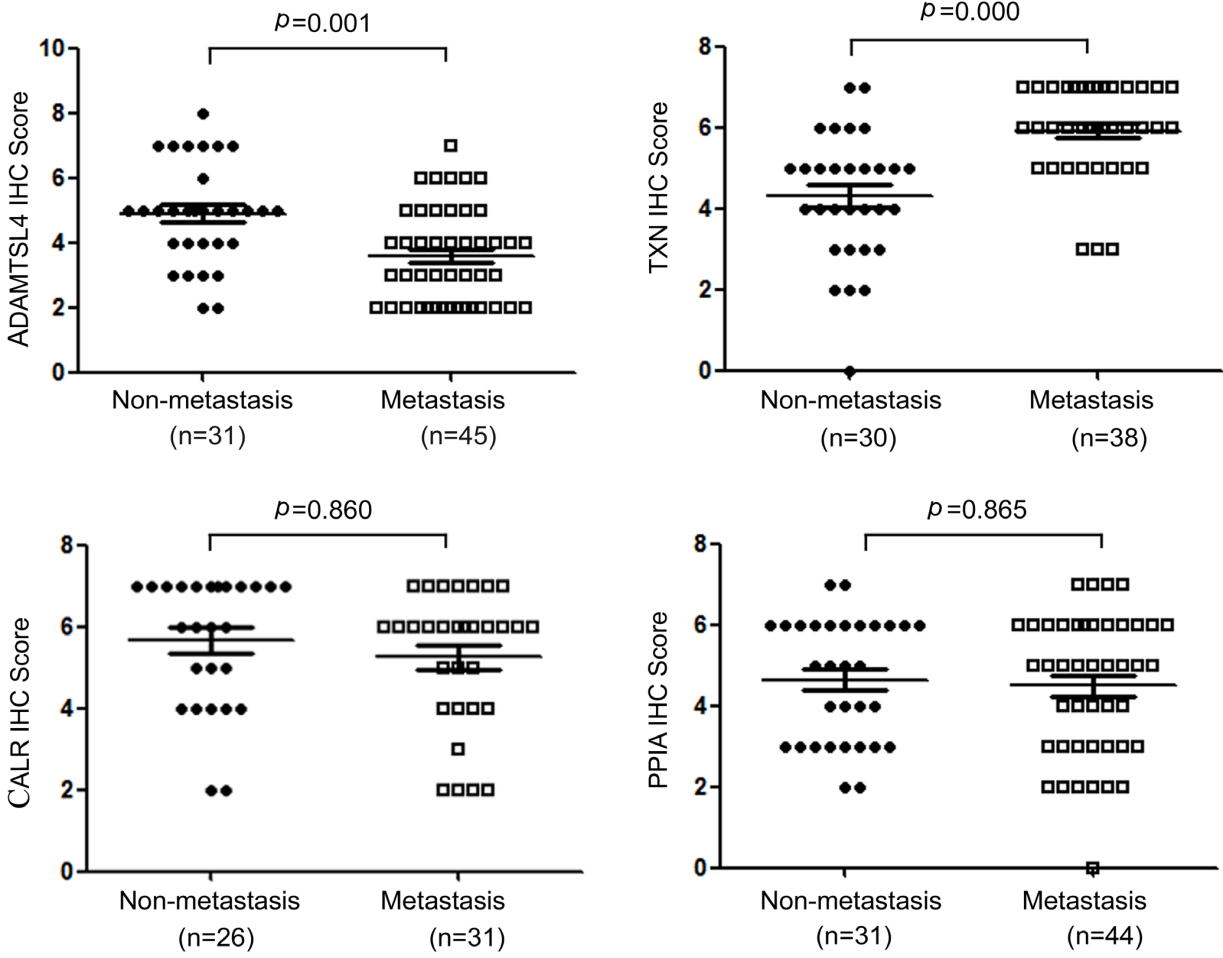
Statistical analysis of the expression levels for ADAMTSL4, TXN, CALR and PPIA in non-metastasis and metastasis NPC tissue specimens

The immunohistochemistry was carried out to further measure the expression levels of the four DEPs (ADAMTSL4, CALR, PPIA and TXN) in a cohort of NPC tissue specimens from metastatic and non-metastatic NPCs. As shown in Figure 2-3 and Table 1, the expression level of ADAMTSL4 was significantly lower in the metastasis NPCs than non-metastasis NPCs (*P* = 0.001), but the expression level of TXN showed an opposite behavior (*P* = 0.000). This result also supports our findings in the NPC cells. However, the expression levels of CALR and PPIA showed no significant differences in the metastasis and non-metastasis NPCs (*P* > 0.05). To be specific, the analysis combined with the clinical data (Table 2-3) indicated that the expression level of ADAMTSL4 showed negative correlations with the nasopharyngeal carcinoma lymph node metastasis (*P* = 0.002), distant metastasis (*P* = 0.020) and clinical staging (*P* = 0.006). The expression level of TXN protein in NPC tissues with lymph node metastasis (*P* = 0.000) and distant metastasis (*P* = 0.026) were higher than those in NPCs without metastasis.

**Table 1:** Expression of ADAMTSL4, TXN, CALR and PPIA in NPCs

		Expression level		
	n	Low (0-4)	High (5-8)	*P*
**ADAMTSL4**				
Non-metastatic NPC	31	11	20	0.001*
Metastatic NPC	45	33	12	
**TXN**				
Non-metastatic NPC	30	15	15	0.000*
Metastatic NPC	38	3	35	
**CALR**				
Non-metastatic NPC	26	7	19	0.860
Metastatic NPC	31	9	22	
**PPIA**				
Non-metastatic NPC	31	14	17	0.865
Metastatic NPC	44	19	25	

*P < 0.05, by χ ^2^ test, Non-metastatic NPC *vs*. Metastatic NPC.

**Table 2:** Relationship between ADAMTSL4 expression and clinicopathological factors in NPCs

Variable		Expression level		*P*
	n	Low(0-4)	High(5-8)	
**Age**				
<50 years	47	28	19	0.706
≥50 years	29	16	13	
**Gender**				
Male	55	29	26	0.140
Female	21	15	6	
**Primary tumor(T)stage**				
T1-2	24	11	13	0.148
T3-4	52	33	19	
**Lymph node(N) metastasis**				
N0	32	12	20	0.002*
N1-3	44	32	12	
**Distant metastasis(M)**				
M0	56	28	28	0.020*
M1	20	16	4	
**Clinacal stage**				
I+II	15	4	11	0.006*
III+IV	61	40	21	

*P <0.05 by χ^2^ test, N0 *vs*.N1-3; M0 *vs*.M1. TNM stage I-II *vs*. III-IV.

**Table 3:** Relationship between TXN expression and clinicopathological factors in NPCs

Variable		Expression level		*P*
	n	Low(0-4)	High(5-8)	
**Age**				
<50 years	43	12	31	0.725
≥50 years	25	6	19	
**Gender**				
Male	46	13	33	0.628
Female	22	5	17	
**Primary tumor(T)stage**				
T1-2	22	3	19	0.097
T3-4	46	15	31	
**Lymph node(N) metastasis**				
N0	31	15	16	0.000*
N1-3	37	3	34	
**Distant metastasis(M)**				
M0	51	17	34	0.026*
M1	17	1	16	
**Clinacal stage**				
I+II	13	3	10	0.758
III+IV	55	15	40	

*P<0.05 by χ^2^ test, N0 *vs*.N1-3; M0 *vs*.M1.

### ELISA analysis of ADAMTSL4 and TXN in NPC patients

**Figure 4:**
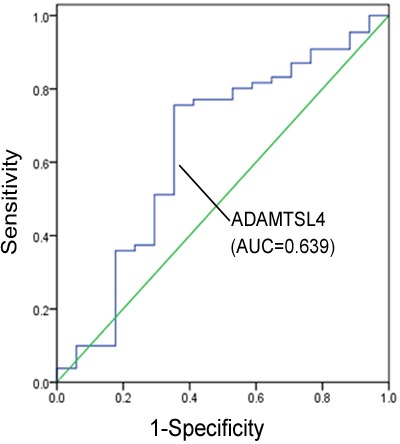
Receiver operating characteristic (ROC)curve for ADAMTSL4 discriminating metastasis NPC patients (*n* = 131) from non-metastasis NPC patients (*n* = 17)

According to the results of Western blotting and immunohistochemistry analyses, the expression levels of ADAMTSL4 and TXN were subsequently investigated in serum of 17 NPC patients without metastasis and 131 NPC patients with metastasis. Commercial ELISA kit was used for these two type of patients and it has identified that the serum ADAMTSL4 levels were much lower in metastasis NPC patients than those in non-metastasis NPC patients (*P* = 0.045), whereas the serum TXN levels were similar for the NPC patients with and without metastasis (*P* = 0.142) (Table 4). The ability of ADAMTSL4 to discriminate metastasis NPC patients from non-metastasis NPC patients was evaluated by plotting the receiver operating characteristic (ROC) curve, which showed an AUC value of 0.639 (Figure 4). Using 5.58 ng/ml as the cutoff value that produced the optimal detection performance, we found that the sensitivity and specificity values for ADAMTSL4 were 75.6% and 64.7%, respectively.

**Table 4:** Serum levels of ADAMTSL4 and TXN in non-metastasis and metastasis NPC patients and the correlation of proteins with clinicopathological factors in NPCs

Variable	n	ADAMTSL4 (ng/ml)	*p*	n	TXN (ng/ml)	*p*
**Age**						
<50 years	81	4.35±2.19	0.681	76	25.19(18.84-41.99)	0.731
≥50 years	67	4.18±2.60		64	32.54±18.38	
**Gender**						
Male	109	4.50±2.45	0.051	101	28.07(19.29-43.53)	0.106
Female	39	3.64±2.07		39	27.97±17.25	
**Primary tumor(T)stage**						
T1-2	54	4.68±2.49	0.114	50	37.20±19.43	0.013*
T3-4	94	4.04±2.29		90	24.19(16.90-38.56)	
**Lymph node(N) metastasis**						
N0	17	5.36±2.73	0.045*	14	25.19±13.66	0.142
N1-3	131	4.13±2.30		126	27.56(18.78-43.02)	
N0-1	67	5.16±2.36	0.000*	61	33.09±20.14	0.911
N2-3	81	3.53±2.13		79	25.59(19.23-42.46)	
**Distant metastasis(M)**						
M0	138	4.31±2.40	0.328	130	26.23(18.87-43.02)	0.275
M1	10	3.65±2.21		10	25.58±14.53	
**Clinacal stage**						
I+II	28	5.63±2.61	0.001*	26	37.58±22.11	0.145
III+IV	120	3.95±2.21		114	25.63(18.71-41.19)	

*P< 0.05 by Student *t* test or Wilcoxon test.

ADAMTSL4: N0 *vs*.N1-3, N0-1 *vs*.N2-3, Clinacal stage I+II *vs*. III+IV.

TXN: T1-2 *vs*.T3-4

## DISCUSSION

In the process of cancer progression, the proteins expression levels can be altered by the genetic mutations, and accordingly affect the tumor growth and metastasis [19]. Furthermore, only the extracellular proteins secreted by the tumor cells or tissues are likely to enter blood or other body fluids and also achieve a “measurable” concentration. Thus, the research on the secretory proteins would be beneficial to discover the novel serum tumor biomarkers.

In this study, we firstly used the serum-free culture method to collect the cell culture supernatant. After ultrafiltration concentration, the supernatant was analyzed by Western blotting. It revealed that all these five targets (GSN, ADAMTSL4, CALR, PPIA and TXN) can be secreted by the NPC 6-10B and 5-8F cells, which provided an important basis for our following studies to identify the serum tumor biomarkers of NPC. However, the secretion mechanisms for these proteins still need to be further clarified.

In order to find more specific serum tumor biomarkers, Western blotting and immunohistochemistry were conducted to evaluate the expression levels of the five proteins in NPC cells and NPC tissues, respectively. Combining with our previous studies, we found that only ADAMTSL4 and TXN presented the consistent results in the experiments of iTRAQ-tagging combined with 2D LC-MS/MS, qRT-PCR, Western blotting and immunohistochemistry. CALR and PPIA presented the consistent results in the experiments of iTRAQ, qRT-PCR and Western blotting, While GSN only presented the consistent results in the experiments of iTRAQ and qRT-PCR. The process of protein biosynthesis includes transcription, translation, and post translational modification. The level of mRNA only reflects the transcriptional process, while the process of post translational modification is changeful and unpredictable. Thus, it is possible that mRNA levels are not consistent with protein levels [20]. In addition, because different regulation mechanisms of protein (such as synthesis and degradation) *in vivo* are complex, the result of *in-vitro* studies cannot fully reflect the true condition *in vivo*, the result of the experiments *in vivo* and *in vitro* may different.

ADAMTSL4, discovered in 2003 [21], is a glycoprotein, belongs to the family of ADAMTS-Like protein (A Disintegrin And Metalloproteinase with Thrombospondin Motifs Like) [22], and also known as Thrombospondin Repeat Containing Gene 1(TSRC1) [21]. It has been reported that ADAMTSL4 can regulate cell apoptosis and participate widely in cell biology by interacting with cathepsin B (CTSB), muscle fibril protein 1 (FBN1), etc [21, 23–26]. In addition, Jianping Liu and his colleagues [27] found that the overexpression of ADAMTSL4 could facilitate the tumor necrosis factor (TNF) induced OV-90 cells (ovarian cancer cells) apoptosis. It suggests that ADAMTSL4 is a possible tumor suppressor gene. Our previous and present studies showed that both ADAMTSL4 protein and mRNA were significantly down-regulated in high metastatic NPC 5-8F cells compared with non-metastatic NPC 6-10B cells. Furthermore, the result of immunohistochemistry analysis indicated that the expression level of ADAMTSL4 was highly correlated with the NPC lymph node metastasis, the distant metastasis and the clinical staging. Thus, it is reasonable to identify ADAMTSL4 as a tumor suppressor gene for NPC preventing the tumor progression and metastasis, which is well consistent with its positive regulation apoptosis function as indicated above.

Thioredoxin (TXN), the other DEP in NPC cells, is a ubiquitously expressed protein with the molecular mass of 12 kDa, and originally described as the hydrogen donor for ribonucleotide reductase in Escherichia coli [28]. Numerous subsequent studies demonstrated that TXN plays a role in a wide variety of redox dependent cellular processes, such as gene expression, signal transduction, cell growth and apoptosis [29–30]. Various TXN targets and interacting molecules have been identified, for example, by modulating the DNA binding activity of transcription factors, including nuclear factor-κ B, p53, glucocorticoid and estrogen receptors [31–32]. The association of TXN with cancers was analyzed by immunohistochemistry using anti-TXN antibody. Results showed that the expression of TXN was detected in a number of human cancer tissues, including liver, colon, pancreas and uterine cervix indicating the possible involvement of TXN in the process of oncogenesis [32]. To be specific, it was reported that TXN could promote tumor growth and inhibit apoptosis in liver cancer, lung cancer, prostate cancer and other various cancers [33]. In addition, it was showed that TXN was over-expressed in human breast carcinoma tissues and the expression level was correlated with tumor grade [34]. In our studies, both TXN protein and mRNA were significantly up-regulated in 5-8F cells compared with in 6-10B cells. Moreover, the expression level of TXN protein in NPC tissues with lymph node metastasis and distant metastasis was higher than that in NPC tissues without metastasis, which suggested that TXN may play an important role in promoting NPC metastasis. However, due to the less NPC patients with clinical stage I and II (*n* = 13) than the patients with stage III and IV (*n* = 55), the correlation of TXN expression with clinical stages need to be further certified.

Based on the translational medicine, we hope to obtain practical indicators from clinical accessible human blood or other body fluids for early diagnosis and evaluation of the prognosis of NPC patients. According to our previous ELISA analysis, on the serum ADAMTSL4 and TXN levels in NPC patients without any therapy prior to the diagnosis, it was found that the serum ADAMTSL4 levels in non-metastasis NPC patients were higher than those in metastasis NPC patients, whereas the serum TXN levels in non-metastasis and metastasis NPC patients were not significantly different. Further analysis combined with clinical data showed that serum ADAMTSL4 levels were much higher in patients with Lymph node metastasis of N0/N1 than those with N2/N3, and also much higher in early NPC patients than advanced NPC patients, which further confirmed the negative correlation of serum ADAMTSL4 levels with lymph node metastasis and clinical stage of NPC. But we have not identified the association between the serum ADAMTSL4 levels and the NPC distant metastasis because few NPC patients with distant metastasis (*n* = 10) were diagnosed. Because most NPC patients were suffering from the cervical lymph node metastasis, few NPC patients were diagnosed without metastasis, and thus the value of AUC was not ideal. At follow-up research, we will continue to increase the samples and make the results more convincing.

In conclusion, we found that the five targets, including GSN, ADAMTSL4, CALR, PPIA and TXN, could be secreted by both NPC-6-10B and 5-8F cells into extracellular. It provides an important basis for discovering serum markers of NPC metastasis. The Western blotting and immunohistochemistry analyses verified that in these five proteins, ADAMTSL4 and TXN were closed related to the metastasis of NPC. ADAMTSL4 inhibited the metastasis while TXN facilitated it. Thus, these two proteins might be the important molecular biomarkers for predicting NPC metastasis and prognosis. The ELISA analysis demonstrated that the serum ADAMTSL4 level was highly associated with the NPC lymph node metastasis and the clinical stage. The present studies suggest for the first time that ADAMTSL4 could be a potential serum tumor marker for predicting the NPC metastasis. However, the specific molecular mechanisms and signaling pathways are unclear and need to be further investigated.

## MATERIALS AND METHODS

### Paraffin tissues and serum specimens from NPC patients

The 98 paraffin-embedded NPC tissue specimens (44 NPCs without metastasis and 54 NPCs with metastasis) were obtained between Jan. 2007 and Dec. 2013 from the Xiangya Hospital of Central South University (Changsha, China) and the First Hospital of Chengzhou City (Chengzhou, China) at the time of diagnosis prior to any therapy. All serum specimens (17 NPCs without metastasis and 131 NPCs with metastasis) were obtained between Jun. 2014 and Apr. 2015 from the Xiangya Hospital of Central South at the time of diagnosis before any therapy. On the basis of the 1978 WHO classification [35], all tumors were histopathologically diagnosed as poorly differentiated squamous cell carcinomas (WHO type III). The clinical stage of all the patients was classified according to the 1992 NPC staging system of China [36]. All procedures performed in studies involving human participants were in accordance with the ethical standards of the institutional and/or national research committee and with the 1964 Helsinki declaration and its later amendments or comparable ethical standards. This study was approved by the Hunan Ethics Committee of Xiangya Hospital of Central South University(Changsha, China).

### Cell culture and harvesting of cell extracts and secreted/shed proteins from conditioned medium

High metastatic NPC 5-8F and non-metastatic NPC 6-10B cell lines were cultured in RPMI-1640 medium (GibcoLife Technologies) supplemented with 10% festal bovine serum(FBS, GibcoLife Technologies) in a humidified chamber with 5% CO_2_ at a temperature of 37°C. On the one hand, cells were lysed in RIPA lysis buffer, containing 7 M urea, 2 M thiourea, 65 mM dithiothreitol and 0.1 mM phenylmethylsulfonyl fluoride (ComWin Biotech) to extract cell extracts. On the other hand, after growing to approximately 70% confluence in 250-mm culture dishes (NEST Biotechnology), cells were washed with 10 ml serum-free medium (RPMI-1640 medium) for three times, and incubated in serum-free medium at 37 °C for 24 h. After incubation, the conditioned medium was collected and centrifuged at 900 × g for 10 min to eliminate suspended cells. The supernatants were concentrated and desalted using Amicon Ultra-15 tubes (molecular mass cutoff, 3KDa; Millipore), followed by addition of proteinase inhibitor cocktail (1 mM phenylmethylsulfonyl fluoride (PMSF), Auragene Bioscience). The concentration of the proteins in cell extracts and supernatants were determined using the BCA protein assay reagent (Tiangen biotechnology). The proteins in cell extracts and collected conditioned media were then stored at −80 °C until use.

### Western blotting

Proteins (45μg) in cell extracts and conditioned media were separated by SDS-PAGE, transferred onto polyvinylidene difluoride (PVDF) membranes (Millipore). The membranes were blocked with 5% nonfat dry milk for 2h at room temperature, and incubated with primary antibodies against GSN (1:1500, Proteintech, 11644-2-AP), PPIA (1:2000, Proteintech, 10720-1-AP), TXN (1:200, Santa Cruz, sc-20146), ADAMTSL4 (1:500, Proteintech, 11644-2-AP), CALR (1:1000, Abcam, ab22683) overnight at 4 °C, followed by incubation with horseradish peroxidase-conjugated secondary antibody (1:10000, Abcam) for 1 h at room temperature. The signal was visualized with Luminata Crescendo Western HRP Substrate (Millipore) and quantitated by densitometry using Image Quant image analysis system (Storm Optical Scanner, Molecular Dynamics, Sunnyvale, CA).

### Immunohistochemistry and staining evaluation

Immunohistochemistry was performed on formalin-fixed and paraffin-embedded tissue sections using a standard. Briefly, after deparaffinization and rehydration, 4 μm of tissue sections were incubated in 3% hydrogen peroxide for 10 min at room temperature to quench endogenous peroxidase activity and immersed in boiling ethylenediamine tetraaceticacid (EDTA) buffer (pH 9.0) for antigen retrieval. After incubating in 5% normal bovine serum albumin for 10 min to block non-specific conjugation, the sections were incubated overnight at 4 °C with four primary antibodies: anti-ADAMTSL4 (1:100), anti-CALR (1:200), anti-PPIA (1:200) and anti-TXN (1:100), and then incubated again with secondary antibody for 15 min at 37 °C followed by avidin-biotin peroxidase complex according to themanufacturer's instructions. Finally, the sections were incubated with 3′, 3′-diaminobenzidine (DAB) until a brown color developed and counterstained with Harris’ modified hematoxylin. In negative controls, primary antibodies were omitted.

The staining intensities of the four proteins were evaluated independently by two pathologists. A quantitative score was performed by adding the score of staining extent and the score of staining intensity for each case to assess the expression levels of the proteins. The intensity of staining was categorized: negative as 0, bordering as 1, weak as 2, moderate as 3 and strong as 4. The percentage of stained cells was categorized: no staining was scored as 0, 1-25% as 1, 26~50% as 2, 51~75% as 3 and 76-100% as 4. The staining score for each tissue was calculated by adding the area score and the intensity score. A combined staining score of ≤4was considered to be low expression while a score of > 4 was considered to be high expression.

### ELISA

Serum levels of ADAMTSL4 and TXN were measured using an ELISA kit according to the manufacturer's protocol (CUSABIOS systems:CSB-EL001320HU/ CSB-E09728h). Briefly, 100 μL of serum was added into microplate wells and incubated for 2 h at 37 °C. Then, the liquid of each well was removed (no washing) and 100 μL of biotin-conjugated anti-ADAMTSL4 (or TXN) anti-body was added into each well and incubated for 1 h at 37 °C. After washing wells with 200 μL wash buffer for three times, 100 μL of HRP-avidin was added into each well and plates were incubated for 1 hat 37°C. After repeating the washing step, 90 μL of TMB substrate was added into each well and plates were incubated for 20 min at 37°C for color development. Finally, reactions were stopped by adding 50 μL of 1 M sulfuric acid and the optical density of each well in the microplate was measured at 450 nm using a microplate reader(Tecan). Three repeated wells of each serum specimen in ELISA were performed and the result was the average of the three repeated.

### Bioinformatics analysis

GO analysis, miRNAs analysis, pathway and PPI analysis of the 5 DEPs(GSN, ADAMTSL4, CALR, PPIA, TXN) were annotated by using CapitalBio MAS 3.0 (http://bioinfo.capitalbio.com/mas3/).

### Statistical analysis

All statistical analysis was conducted using SPSS17.0 software. Results are expressed as mean(standard deviation, SD) or median (interquartile range, IQR) for the continuous variables. The differences of the ADAMTSL4, CALR, PPIA and TXN protein expression between metastasis and non-metastasis NPC tissues were analyzed using χ ^2^ test. Serum levels were compared using the Student *t* test or Wilcoxon test. Receiver operator characteristic (ROC) curves were constructed by plotting sensitivity *versus* (1-specificity). *P* values less than 0.05 were considered to be statistically significant.

## SUPPLEMENTARY MATERIALS FIGURE


