# Supplementary material for: Correlation of five secretory proteins with the nasopharyngeal carcinoma metastasis and the clinical applications
Source: Oncotarget. 2017 Jan 18;8(17):29383–94. doi: 10.18632/oncotarget.14725 (PMC5438738; doi:10.18632/oncotarget.14725)
Supplement: Supplementary file 1 [file oncotarget-08-29383-s001.pdf]

Correlation of five secretory proteins with the nasopharyngeal carcinoma metastasis and the clinical applications

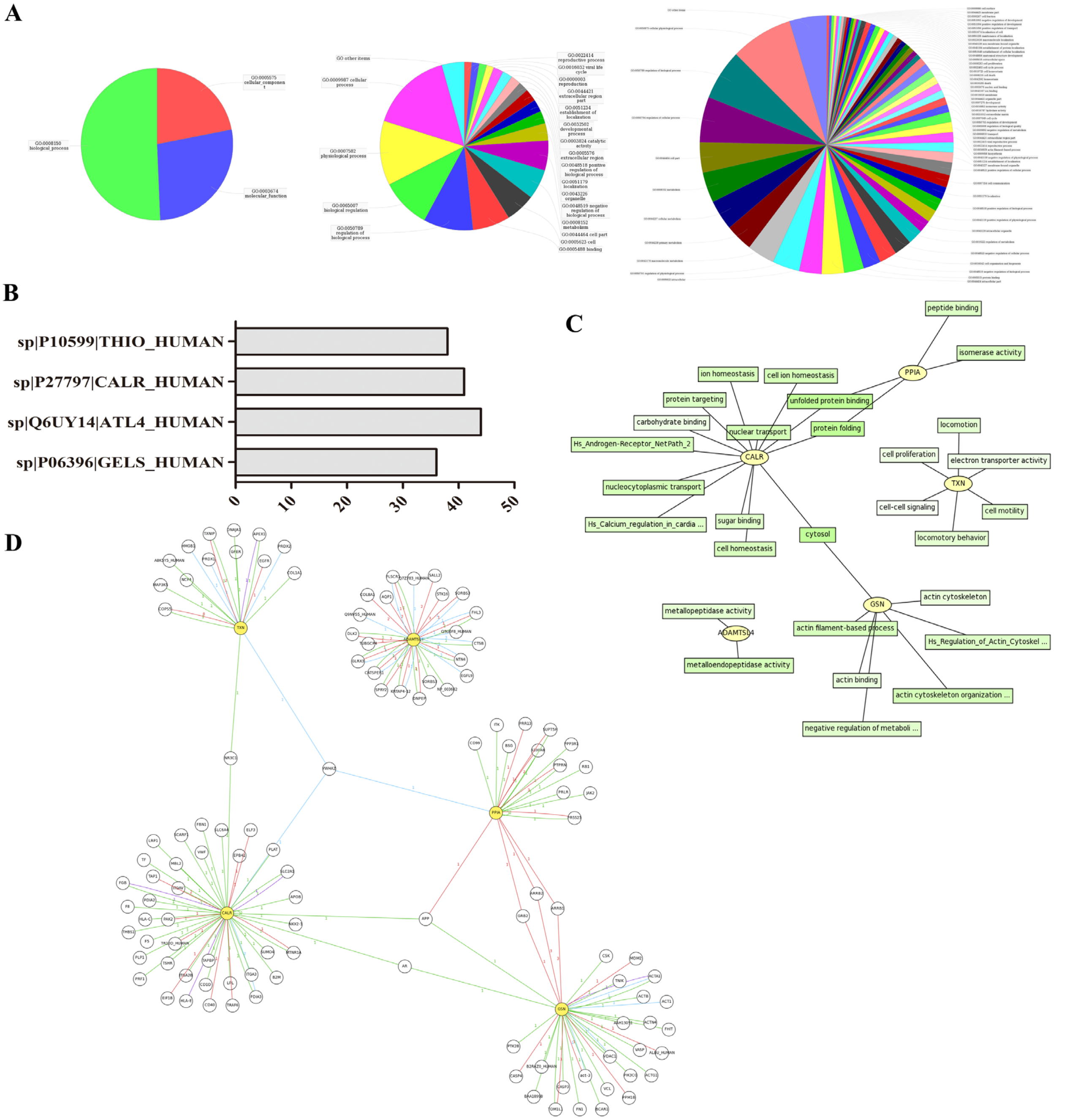

Supplementary Figure S1: Bioinformatic analysis of the 5 DEPs in NPC development process.(A)GO analysis of the 5 DEPs by dividing into molecular function,biological process and cellular component;(middle)further dividing into particular GO;(right) further dividing into more particular GO. The area of the pie represented proportion of every GO.(B) Predicted miRNAs of the differential genes, Columns, means different genes; bars,number of miRNAs.(C) GenMAPP pathway analysis showed that the differentially proteins significantly enriched in all kinds of signaling pathways.(D) PPI networks of differentially-expressed genes. Lines of different colors represent different original database.The yellow origin represents the afferent protein. The light blue line represents the MINT database, the green line represents HPRD database, the red line represents IntAct database, the purple lines represent DIP database.Count exist amount of two different proteins among the same interaction of the same database.
